# Social Task Regulation in the Dimorphic Ant, *Pheidole pallidula*: the Influence of Caste Ratio

**DOI:** 10.1673/031.010.0301

**Published:** 2010-02-18

**Authors:** Grégory Sempo, Claire Detrain

**Affiliations:** Unit of Social Ecology, CP 231, Université libre de Bruxelles, Bld du Triomphe, 1050 Brussels, Belgium

**Keywords:** division of labor, social regulation, behavioral repertoire, colony composition, *Pheidole*, *Periplaneta americana*

## Abstract

We investigated whether the physical castes of the dimorphic ant *Pheidole pallidula* (Nylander) (Hymenoptera: Formicidae), are involved in determining within-nest activities and how their social investment in everyday tasks is influenced by large changes in the colony's caste ratio. Although the large-headed majors are morphologically distinct from minors, they are similar in size, exhibit similar behavioral repertoires and carry out nearly the same tasks as minors. Changes, even large ones, in the colony's caste ratio have no significant effect on the repertoire size of either caste. Majors do not compensate for the depletion of minors by expanding their repertoire or increasing their activity level. Instead of being an idle stand-by caste as suggested for other *Pheidole* specie s, *P. pallidula* majors are nearly as totipotent as minors. Moreover, their performance rate of social behaviors is remarkably high and constant regardless of the colony caste ratio. Such high investment of the major caste helps the colony to keep social behaviors at a baseline even in colonies undergoing large demographic changes. Alternative schemes of social regulation in polymorphic ant species are discussed. A possible methodological bias accounting for between-species differences in the level of majors' specialization is described.

## Introduction

A common way of organizing work in insect societies is through the division of labor in which individuals consistently perform a subset of tasks for periods ranging from a few days to their whole lives. At the colony level, the simultaneous performance of different tasks by different groups of specialized individuals is assumed more efficient than if tasks were performed sequentially by unspecialized πindividuals (Oster and Wilson 1978; Jeanne 1986; Gordon 1989; Robinson 1992; Bourke and Franks 1995).

Task specialization is known to be influenced by the age of workers, their morphology, their genetic background and their individual experience in bees and in ants (for bees, see Robinson and Page 1988; Seeley 1995; Dreller and Page 1999; Huang and Robinson 1999; Fewell and Bertram 2002; Hurd et al. 2007; and for ants, see Gordon 1989, 1996; Hölldobler and Wilson 1990; Theraulaz et al. 1998; Beshers et al. 1999; Tripet and Nonacs 2004; Heredia and Detrain 2005; Seid and Traniello 2006; Ravary et al. 2007). Several studies have shown that division of labor between workers is a dynamic phenomenon: the species-specific behavioral profile of each caste can be altered by day-to-day or seasonal changes in the colony composition due to predation, competitive pressure or changes in environmental resources (Davidson 1978; Sorensen et al. 1984; Johnston and Wilson 1985; Wheeler 1991; Aarab and Jaisson 1992; Schmid-Hempel 1992; Tschinkel 1993; Gordon 1996; Passera et al. 1996). In order to maintain colony organization at a high efficiency level even after large perturbations in its caste ratio, one colony should ideally be able to fill-in for missing individuals. Over a long time-scale, a colony can increase the production of new adults belonging to the depleted caste through an adaptive demography response (Calabi and Traniello 1989; Passera 1977; Passera et al. 1996). Over a shorter time-scale, a more flexible response can be achieved by the behavioral plasticity of individuals that perform tasks considered atypical for their caste (Wilson 1980, 1983, 1984, 1986; Detrain and Pasteels 1991; Cassill and Tschinkel 1999) and/or change their activity rate (Wilson 1984; Brown and Traniello 1998).

Regarding dimorphic ant species, physical castes are expected to differ in their behavioral capabilities. From an ergonomic perspective (Oster and Wilson 1978) it is assumed that the numerically dominant minor caste consists in “generalists,” workers that take care of all tasks necessary to colony development. Contrastingly, majors are “specialists” that perform only a subset of the minors' behavioral repertoire ([Bibr bibr63][Bibr bibr64], 1984; Calabi and Traniello 1989; Brown and Traniello 1998). Species of the ant genus *Pheidole* typically possess such dimorphic workers: minor workers perform most tasks within the nest and forage while majors, with their disproportionately large head, are specialized for colony defense, seed milling and food storage (Wilson 2003). With its worldwide distribution and with its diversity (more than 900 described species, see Bolton 1995; Wilson 2003), *Pheidole* has become a key genus for investigating the adaptive nature of caste morphology among species living under different ecological conditions. Furthermore, the diversity of *Pheidole* has provided a framework to investigate questions concerning the evolutionary ecology of morphological variation as well as the interplay between morphological and behavioral specialization (Pie and Traniello 2006; Moreau 2008).

Several authors have suggested that the breadth of the behavioral repertoire of minors has been a stabilizing factor, buffering the need for morphological specialization in specific tasks such as brood care and nest construction (Bolton 1995; Pie and Traniello 2006; Moreau 2008). Conversely, the distinct head morphology of majors appears related to their behavioral specialization for a limited number of tasks. In conditions of highly disturbed caste ratio, majors may enlarge their repertoire size and/or increase their performance rate of some behaviors to compensate for a depletion of the minor caste (Wilson 1984, 1986; Calabi and Traniello 1989; Brown and Traniello 1998; Burkhardt 1998). However, this behavioral flexibility of majors needs to be examined by separating possible sampling size effects (Sempo and Detrain 2004) from actual changes in their behavioral capacities.

The close relationship between caste morphology and behavioral specialization in the *Pheidole* genus has been highlighted on several species of the New World, which is assumed to be the cradle of this “hyperdiverse” genus (Moreau 2008). The Old World species, *Pheidole pallidula* (Nylander) (Hymenoptera: Formicidae), offers a contrasting picture of division of labor. With a degree of morphological specialization similar to New World species, *P. pallidula* majors show an unexpectedly large behavioral repertoire: they are involved in defense (Detrain and Pasteels 1992) as well as foraging (Detrain and Pasteels 1991; Detrain and Deneubourg 1997), food storage (Lachaud et al. 1992) and brood care (Sempo and Detrain 2004). With such an extended behavioral repertoire of both minor and major castes, whether and how does social regulation occur in *P. pallidula* colonies? Would majors further enlarge their behavioral repertoire following deep changes in the colony's caste ratio? If their behavioral repertoire remains unchanged, would majors change their activity rate or their engagement in social behaviors within the nest?

## Methods

### Collection and rearing of colonies

*P. pallidula* colonies were collected on rocky calcareous in Gonfaron (France). Colonies were composed of one queen and 4,500 to 5,500 workers. Eggs, nymphs and larvae belonging to all three instars of *P. pallidula* were present in the colony. The worker caste consisted of 10 to 12% majors, which was a percentage close to that reported in the field by Passera (1977).

In the laboratory, colonies were housed in moistened nest tubes (length: 16 cm; diameter: 1.5 cm) placed in plastic trays (length: 30 cm; width: 20 cm; height: 4 cm). The plastic trays' walls were coated with Fluon to prevent ants from escaping. In the foraging area, ants had permanent access to water and brown sugar solution (concentration: 1M), and they received one dead cockroach (*Periplaneta americana*) per week. Colonies were maintained in the dark at 28°±1°C which is a temperature that maximizes the production of major workers among larvae (Passera 1974).

### Definition of behavioral acts

Inner-nest behaviors possibly performed by *P. pallidula* workers were listed based on preliminary observations (Sempo and Detrain 2004) and data reported for other *Pheidole* species (Wilson 1976a, 1984; Calabi et al. 1983; Patel 1990; Brown and Traniello 1998).

Queen care was excluded as a possible behavior because all tested colonies were queenless. There were 38 remaining behaviors included in the *P. pallidula* repertoire. These behaviors were classified in the following main categories: egg care, care of first and second instars larvae, care of third instar larvae, care of pupae, cleaning the nest, self-grooming, allogrooming, antennal contacts and trophallaxies. Ants were arbitrarily considered inactive if they did not move or engage in any tasks for at least 5 seconds.

### Worker behavior in colonies with modified caste ratio

Experimental colonies were reared in nests (4 cm × 4 cm × 0.2 cm) dug in a plaster of Paris layer poured in a polyethylene box (15 cm × 10 cm × 7 cm). A red glass plate covered the top of the nest to create the darkness necessary for the ant settlement while allowing observation of the brood and of the ants' behavior within the nest.

From 16 mother colonies, 56 queenless colonies were made consisting of 10, 25, 50, 60, 75 or 90% of majors. For each of the tested caste ratios, excepting 10 and 90% of majors, four genetically distinct colonies were used. For extreme caste ratios (10 and 90%) a higher number of colonies (n=20) were required: these were made from 16 distinct mother colonies with four of them being used twice. The use of 16 additional colonies containing 10% and 90% implies that for these caste ratios only, 4 pairs of colonies come from the same 4 mother colonies.

Each of the colonies were made by picking up ants from all parts within the mother nest as well as from its foraging area. After allowing workers to acclimate to their experimental nest for 24 hours, brood was deposited in front of the nest entrance allowing workers to retrieve it into the nest chamber. The amount of brood introduced in each experimental nest was arbitrarily fixed to 15 eggs, 15 larvae of first and second instars, 15 third instar larvae, 13 minor pupae and 2 major pupae.

The proportion of majors among workers staying within the nest was significantly correlated to their proportion among the 100 ants introduced in the setup (Spearman rank correlation: *rs* = 0.92, *n* = 260, *P* < 0.0001). Since the fraction of majors introduced in the setup is highly representative of the fraction of majors actually present within the nest, it is used as the X-axis unit for all figures presented hereafter.

The first set of behavioral observations took place two days after the introduction of brood. This time allowed workers to organize brood spatially and to re-allocate tasks within the new experimental set-up. For each colony, five sessions of behavioral observations were carried out at 9a.m., 12p.m., 3p.m., 6p.m., and 9p.m. respectively. Each observation session consisted of scanning the entire colony and recording the behaviors performed by every minor and major workers present within the nest. The behavioral repertoire of each nest was determined by pooling results from these five observation sessions. The nest was covered with a grid network (4cm × 4cm) composed of 16 numbered quadrates (1cm × 1cm) to systematize observations during the scanning.

Four different colonies were used for each tested caste ratio (25%, 50%, 60% or 75% majors). For each of the five observation sessions, the behavior of all individuals in the nest was recorded once through a systematic scanning of all quadrates. Consequently, the proportion of observed individuals belonging to one caste was directly related to their proportion within the colony. Knowing the influence of the sampling size on the estimated behavioral repertoire size (Sempo and Detrain 2004), the number of observations made on the two castes was balanced for the two extreme conditions of caste ratio (10% majors and 90% majors). Therefore, more scanning of the minority caste (i.e. majors in 10 % majors colonies and minors in 90% majors colonies) was performed by observing 20 colonies instead of 4.

To investigate social regulation in colonies with modified caste ratio, how the major caste adjusted its relative investment in social behaviors including brood care, allogrooming, trophallaxies, and social interactions with adults to compensate for the minors' depletion was determined. The “non-social behaviors” category included all other activities such as self-grooming, feeding on an insect, nest maintenance and movement inside the nest. In social hymenoptera, colony size is one factor influencing division of labor with workers being less specialized in small nests than in mature, large colonies (Bourke and Franks 1995; Karsai and Wenzel 1998). To check for such a colony size effect for a normal caste ratio (90%) minors) on *P. pallidula* repertoire size, the frequency distribution of minors' behavior in the experimental colonies (100 ants) was compared to that observed in large, mature nests (around 5,000 ants, from data of Table 1 in Sempo and Detrain 2004).

### Statistical analysis

Means are expressed with standard deviations. Probability values are given for two-tailed tests and the null hypothesis was rejected at p < 0.05. Survival curves were drawn to account for the evolution of the behavioral repertoire size of majors with sampling size. The statistical difference between two survival curves was determined by comparing the slope and the elevation of the regression lines obtained after curve linearization through a natural logarithmic data transformation (Zar 1999). The activity rates between castes were compared using a paired *t*-test. A Spearman rank test was used to test the correlation between the percentage of active individuals and the fraction of majors in the colony. The influence of the colony caste ratio on the performance rate of castes was tested using a Kruskal-Wallis test. The influence of colony caste ratio on the total number of social behaviors performed by both castes was determined by using a one-way ANOVA Test followed by a Tukey-Kramer Multiple Comparisons Test.

## Results

### Social regulation through extended repertoire of majors

**Figure1:**
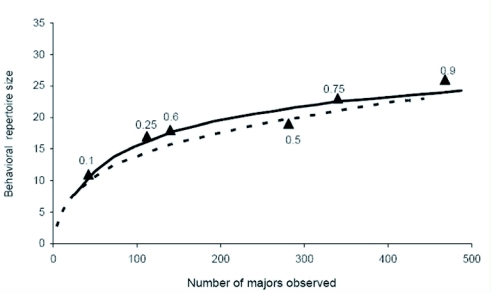
Relationship between the size of the behavioral repertoire of majors and the number of observations made on colonies composed by either 10% of majors (dotted line) or 90% of majors (solid line). Rarefaction curves drawn out of data collected during 100 scanning sessions on 20 colonies and were computed using the *EstimateS* software (Colwell 2000). Sizes of majors repertoire estimated from non-weighted data were given for all tested colony caste ratios (20 scanning sessions on 4 colonies for each colony composition; 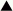 High quality figures are available online.

It is known that the number of observations made on one caste may modify the observer's perception of its behavioral repertoire (Fagen and Goldman 1977; Sempo and Detrain 2004). In these experiments, the sampling size performed on one caste is directly related to its proportion within the nest chamber. Therefore, this estimate of majors' repertoire size is expected to vary with their ratio in the colony. In particular, when majors are few, their repertoire (the number of different behaviors observed) could be underestimated due to rarely occurring behaviors missing. To account for such a possible bias, a rarefaction curve was drawn out from the data set in which majors were the most frequently observed (i.e. colonies with 90% majors). Such a curve predicts how a decrease in sampling size alters an estimate of majors' repertoire. This mathematical tool helps to determine, in colonies with a modified caste ratio, whether changes in majors' repertoire size are due either to changes in sampling size or to genuine changes of their behavioral profile. For each tested colony composition (10, 25, 50, 60, 75 and 90% of majors; Fig. 1), the observed repertoire size of majors fit the value predicted from the rarefaction curve for the same sampling size. The apparent increase of majors' repertoire as a function of their ratio in the colony is thus mainly explained by an increased sampling instead of an enlargement of their repertoire. This statement is confirmed by the wide repertoire displayed by majors even when they account for only 10% of the nest population. Indeed, there is a similarity between the rarefaction curves that were drawn out from the 10% majors and from the 90% majors experiments. After a linearization of these two rarefaction curves using natural logarithmic data transformation, the slopes and elevations of the resulting regression lines were not statistically different (Fig. 1; test of equality of slopes: *df* = 115, *t* = 0.3; p<0.05; test of equality of elevations: *df* = 116, *t* = 0.6, p<0.05). The low level of behavioral specialization of majors is not due to a bias related to the small size of experimental nests (100 ants). Indeed, there is a wide overlap with the behavior expressed by majors that live in large, mature colonies of around 5,000 ants (Table 1). Even in these large colonies, the division of labor does not rest on a highly specialized worker caste performing strictly compartmentalized tasks.

Even though the size of the majors' repertoire is large, the type of acts performed by the majors may differ from those expressed by the minors. Therefore, the behaviors expressed by majors was compared to those expressed by minors in colonies where they, the majors or minors, are the most numerous and thus the more likely to cope with everyday tasks (i.e. the 90% majors and 90% minors colonies, respectively) (Table 1). Regarding social interactions between adults, majors antennated nestmates twice more frequently than minors (Table 1: 0.406 versus 0.243 for antennations done by majors or by minors, respectively). On the other hand, the two castes displayed, at similar frequencies, allogrooming (0.083 for minors vs. 0.078 for majors), regurgitation (0.036 for minors vs. 0.034 for majors) and aggression (0.003 for minors vs. 0.001 for majors). Most differences in frequency values are related to between-caste interactions since the likelihood of one caste interacting with another will depend on the numerical abundance of its partner. For instance, the frequency of allogrooming is higher towards a minor (0.039) in a 90% minor colony than in a 90% major nest (0.004). Moreover, one cannot detect any preferential interactions between individuals of the same caste. An example is that the percentages of allogrooming directed towards a worker of the same caste (For minors: 83%; for majors: 89.1%) is always close to the proportion of this caste in the colony (90% in the two experimental conditions, Table 1).

*P. pallidula* castes also differ in the ways they care for brood both qualitatively and quantitatively. Although most brood-care tasks are performed by majors, they were never seen performing the following 4 brood-care behaviors: lick the eggs; feed first, second and third instar larvae; or carry and roll pupa. Likewise, in the minor caste these behaviors have a very low occurrence (relative frequency < 1%), even though they are important for brood development. Castes, then, differ mainly in a quantitative way since minors are twice more often involved in brood care than majors (relative frequency for minors: 0.205; for majors: 0.104, Table 1).

### Social regulation through increased activity rate

As shown above, the depletion of minors in a colony did not induce qualitative changes in the behavioral repertoire of majors.

**Table 1:**
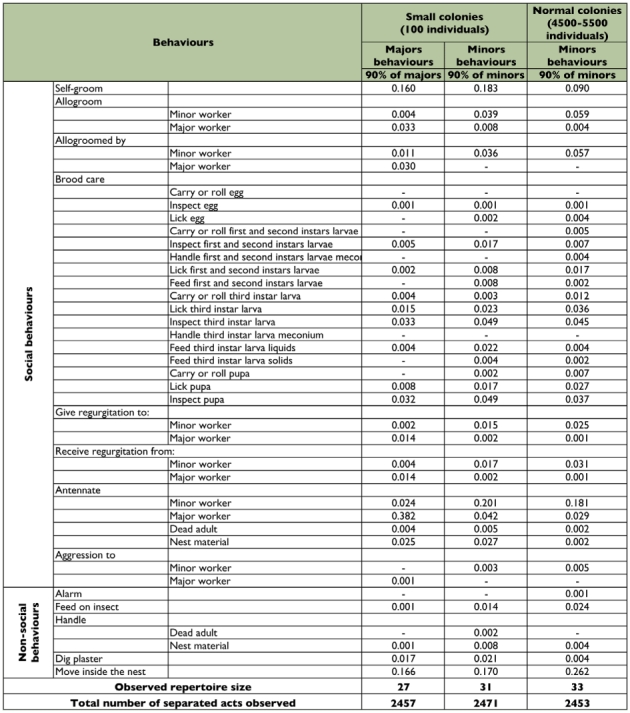
List of the relative frequency of the different behaviours expressed by the numerically dominant caste (90% of the worker population) in small (100 ants) and in normal (around 5,000 ants) *Pheidole pallidula* colonies. These behavioural repertoires were based on a standardized number of around 2,500 observations (data for the normal large colonies are taken from Sempo and Detrain 2004).

**Figure2:**
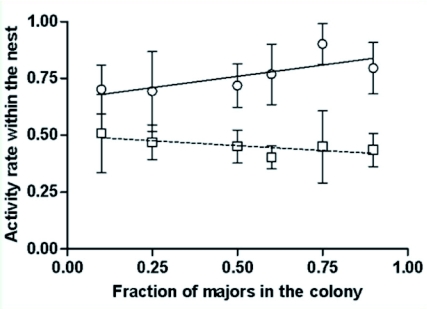
Global activity rare within the nest among minors (circle, solid regression line) and majors (square, dashed regression line) as a function of the fraction of majors in the colony. The fractions of active workers observed during each scanning session were averaged. Bars=mean ± standard deviation, n=20 scanning sessions for each bar. High quality figures are available online.

Nevertheless, social regulation can still occur through quantitative changes of the behavioral profile, by altering either the global activity level of majors or their performance through quantitative changes of the rate of a subset of behaviors. In our study, the activity level of ants within the nest differed between castes. In small, normal colonies (10% majors), minors were involved in more inner-nest tasks than majors were. The activity rate, the percentage of active individuals among all workers of one caste, reached 70.1% ± 10.8 for minors but only 50.8% ± 17.3 for majors (Fig. 2; mean ± SD, *n* = 20; paired t-test: t19 = 7.5, p0.0001). For all of the tested caste ratios, minors continued to display a higher activity rate than majors (Fig. 2). The activity level of minors even increased slightly following their depletion in modified colonies. A significant correlation was observed between the percentage of active minors and the fraction of majors in the colony (Fig. 2; Spearman rank correlation: r = 0.37, n = 56, p < 0.01).

Majors did not significantly change their average activity rate regardless of the composition of the nest population (Fig. 2; Spearman rank correlation: r = -0.23, n = 56, p = 0.09). Indeed, majors always kept a low activity rate ranging between 40% and 50% (Fig. 2). This suggests that *P. pallidula* majors are not a stand-by caste, since they did not shift from a resting to an active status in order to compensate for the depletion of minor workers.

### Social regulation through increased performance of social behaviors

Since the caste ratio did not influence the size of the behavioral repertoire or the global activity level expressed by minors and majors, social regulation could be restricted to a subset of behaviors that matter for social cohesion and ergonomic efficiency. Therefore, 28 social behaviors were considered out of the *P. pallidula* repertoire, including all brood care behaviors and all contacts between adults (antennal, allogrooming, trophallaxies, etc.).

**Figure3:**
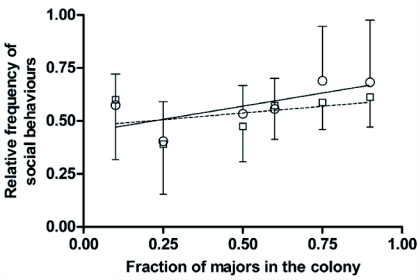
Relative frequency of social behaviors (mean ± standard deviation) performed by minors (circle) and majors (square) as a function of the fraction of majors in the colony. The fractions of social behaviors observed during each scanning session were averaged. Regression line equation: for minors: y=0.249 + 0.445 (solid line); for majors: y= 0.127 + 0.474 (dashed line). High quality figures are available online.

For each caste and for each colony composition, the relative frequency of these social behaviors was calculated. The comparison of these frequencies obtained for the different colony compositions highlights the relative influence of the caste ratio on social behaviors. For minors, no significant changes were observed in the frequency of social behaviors as the proportion of majors increased in the nest (Fig. 3; Kruskal-Wallis test: KW = 6.96, n = 52, p > 0.05). Thus, minors did not compensate for their large depletion in the colony by performing more social behaviors.

Likewise, changes in the colony caste ratio did not significantly alter the performance rate of social behaviors by majors (Fig. 3; Kruskal-Wallis test: KW = 6.14, n = 52, p > 0.05). Consequently, no conclusion can be drawn on a possible compensation of minors' depletion by majors shifting from idle state to higher implication level in social behaviors.

**Figure4::**
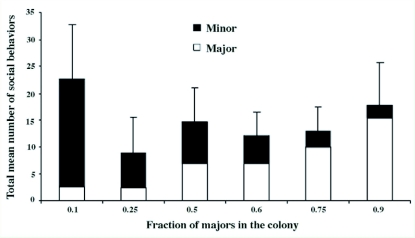
Total number of social behaviors performed by minors (black histogram) and majors (white histogram) as a function of the fraction of majors in the colony. Bars = mean ± standard deviation, n = 20 scanning sessions for each bar. High quality figures are available

Finally, at the colony level, the total social investment of both castes could be influenced by a changed caste ratio. A significant decrease in the total number of social behaviors was observed (ANOVA Test: p <0.0001; Tukey-Kramer Multiple Comparisons Test: p < 0.05 only for comparison between colonies composed of 10% versus 25%, 50%, 60%, 75% or 90% majors) (Fig. 4). As soon as the colony differed from its natural composition (10% majors), the average number of social behaviors observed per scanning was lower and stood between 9 and 17.8 behaviors for colonies composed of 25% majors or more (Fig. 4). The colony was thus able to keep social behaviors at a relatively high level of performance, even after a large depletion of minors.

## Discussion

It is commonly admitted that, in the dimorphic *Pheidole* ant genus, minors carry out nearly all colony tasks while majors are specialized and display only a small part (13% to 19%) of the minors' behavioral repertoire (Wilson 1984; Brown and Traniello 1998).

Besides, it was reported that majors can act as an emergency stand-by caste by enlarging their repertoire in order to compensate for minors depletion due to demographic changes or predation (Wilson 1984). However, the role of majors in social regulation should be reconsidered by paying attention to possible bias due to sampling size. For instance, the largest majors' repertoire (Wilson 1984) was reported for colonies composed of only majors which also implies the largest number of observations (more than 500 majors observed). As this sampling size was up to 20 times higher than on colonies with “natural” caste ratio, the extended repertoire of majors can simply result from the observation of less frequent behaviors. Here it is shown that, in *P. pallidula*, changes in the size of majors' repertoire with colony caste ratio can be explained simply by sampling size effects without invoking qualitative changes in majors behavioral profile (see also Jaisson et al. 1988; Sempo and Detrain 2004). Whatever the caste ratio, the majors' repertoire is strikingly similar to that of minors. Even though they are morphologically specialized with their hypertrophied head and powerful mandibles, *P. pallidula* majors perform all tasks excepting some brood care behaviors and are nearly as totipotent as the minors.

Besides changes in the behavioral repertoire size, social regulation can also take place through an increase in the number of active individuals and/or through an enhancement of individual activity rate. Indeed, inactive individuals constitute a reserve workforce that can assume new tasks depending on colony needs. Those inactive individuals that have no fixed role belong mainly to a transition group intermediate in behavior and age between nurses and foragers (e.g. *Lasius niger* (Lenoir and Ataya 1983) and *Cataglyphis cursor* (Retana and Cerda 1991)). In polymorphic ant species, differences in the activity level between workers are usually related to body size, with minors being more active than majors as shown in *Pheidole guilelmimuelleri* and *P. pubiventris* (Wilson 1984), *P. morrisi* (Patel 1990), *Atta sexdens* (Wilson 1980), *Erebomyrma nevermanni* (Wilson 1986), *Megaponera foetens* (Villet 1990) or *Solenopsis invicta* (Mirenda and Vinson 1981; Sorensen et al. 1984).

Likewise, in the dimorphic *P. pallidula* ant species, inactive individuals are nearly twice more frequent among majors than among minors. Nevertheless, whatever the colony caste ratio, the activity level of *P. pallidula* majors is rather high and quite stable in comparison with other *Pheidole* species (see Wilson 1984; Brown and Traniello 1998). For instance, the ratio of the majors to minors activity rate remains stable at around 0.65 in *P. pallidula*, while it varies between 0.05 and 0.52, depending on the caste ratio in *P. guilelmimuelleri* (Wilson 1984). The relatively high activity level of *P. pallidula* majors cannot be related to a lower degree of morphological specialization as predicted by the ergonomic optimization theory (Oster and Wilson 1978). Indeed, the relative head widths of the minor to the major caste are remarkably similar being 0.39 for *P. guilelmimuelleri* (Patel 1990) and 0.42 for *P. pallidula* (Detrain 1989).

One may question the adaptive value of maintaining a higher proportion of inactive ants among majors since, in *P. pallidula*, resting majors do not participate in social regulation even after large perturbations of the colony caste ratio. Part of the answer lies in the existence of replete majors with distended gaster that account for up to 32% of the total *P. pallidula* majors population (Lachaud et al. 1992) and for nearly 50% of inner-nest majors (personal observation). This sub-caste of majors is mainly located in quiet nest areas far from the nest entrance (personal observation) or near the brood area (Sempo et al. 2006a), does not forage outside the nest or defend it, and is characterized by a very low activity level (Lachaud et al. 1992). Replete majors were also found in other polymorphic ants (*Camponotus* spp. (Wilson 1974; Espadaler et al. 1990; Hasegawa 1993), *Myrmecocystus mexicanus* (Conway 1990), *P. hortensis* (Calabi et al. 1983), *P. morrisi* (Yang 2006) and *Solenopsis invicta* (Mirenda and Vinson 1981)). In polymorphic ants, a distinction can thus be made between two types of inactive majors: (1) idle unspecialized majors that become active due to colony need for an additional workforce as in many *Pheidole* species (Wilson 1984; Brown and Traniello 1998) and (2) replete majors that stay inactive even after large demographic perturbations (as for *P. pallidula* majors), acting as living reservoirs that deaden food shortages.

As shown above, *P. pallidula* societies do not compensate for the depletion of minor caste either by extending the behavioral repertoire of majors or by increasing their activity level. However, among the active individuals, some social regulation may also occur by switching toward activities that are essential for social cohesion (i.e. allogrooming) or for colony survival (i.e. food exchanges and brood care).

For instance, following the removal of one age-class or a traumatic change in the age structure, workers are first and foremost reallocated to social tasks (Lenoir 1979; Meudec and Lenoir 1982; McDonald and Topoff 1985; Calabi and Traniello 1989). Similarly, in polymorphic ants, major workers can show a high sensitivity to a shift in the colony's caste-ratio (i.e. from less than 10% to 90%) majors) as they increased by 15 to 30 times their rate of social behaviors (as shown for three *Pheidole* species in Wilson 1984). Unexpectedly, in *P. pallidula*, the rate of social behaviors performed by majors stayed stable regardless of the colony caste ratio. However, due to their high social investment (≈ 60% of the total number of acts), majors maintain brood care and other essential tasks at a satisfactory baseline level, even in colonies almost deprived of its minor caste.

The ergonomic resiliency of an ant colony relies on its ability to cope with changes occurring over short and long time-scales (Oster and Wilson 1978). In polymorphic ant species, short-term regulation can rely upon the ability of specialized majors to express new, atypical behaviors and/or to increase their performance rate of social activities (Wilson 1984). This short-term behavioral flexibility of the major worker, increasing the rate of activity as well as the behavioral repertoire in case of minor depletion is well described for different species such as *Pheidole guilelmimuelleri* and *P. pubiventris* (Wilson 1984), *P. morrisi* (Patel 1990) and *P. dentata* (Burkhardt 1998; Seid and Traniello 2006). However, this stand-by caste status of majors is not the common rule for all *Pheidole* species. Indeed, *P. pallidula* majors perform an almost full minors repertoire and exhibit a relatively high level of social activities under all circumstances (not only in severe crisis of minors depletion). Secondly, an adaptive demography process may occur to face long-term changes in colony needs and/or environmental constraints (season, predation, etc.). Ideally, the production of majors is expected to be finely tuned to fill-in tasks for which they are specialized, as shown by the increased number of *P. pallidula* majors in the presence of competitors (Passera et al. 1996).

One, however, may notice that, even in similar environmental conditions, there is still a high variability in the caste ratio between colonies of the same species (*P. dentata* (Oster and Wilson 1978), *P. morrisi* (Bhatkar and Whitcomb in Patel 1990; Yang 2006), *P. pallidula* (Passera 1974)). Hence, it could be more advantageous for an ant society, as observed in *P. pallidula*, to dispatch each caste into almost all colony tasks except those which cannot be physiologically or morphologically achieved (for example, in *P. pallidula*: majors do not lay the recruitment trail (Ali et al. 1988; Detrain et al. 1991)).

Without denying the existence of behavioral flexibility among physical castes in ants, this paper has stressed alternative schemes of social regulation. Independent of any qualitative or quantitative changes in the behavioral profile of one caste, other factors, such as a spatial reorganization of ants within the nest, could participate in social regulation. In this respect, differences between castes in aggregative patterns (Sempo et al. 2006a,b) would deserve further investigations in order to be coupled to the efficiency and flexibility of task performance by each worker caste.
